# Supplementary material for: Polyketide synthase gene clusters in the ericoid mycorrhizal fungus Oidiodendron maius: genome-wide prediction, expression profiling, and functional characterization of knockout mutants
Source: Front Plant Sci. 2026 Sep 16;17:1924430. doi: 10.3389/fpls.2026.1924430 (PMC13623903; doi:10.3389/fpls.2026.1924430)
Supplement: Supplementary file 1 [file SupplementaryFile1.zip › Supplementary Materials/Supplementary Folder 1.zip!/Oidma1_AssemblyScaffolds_Repeatmasked/knownclusterblast/cluster50/jgi.p_Oidma1_172946_mibig_hits.html]

| MIBiG Protein | Description | MIBiG Cluster | MiBiG Product | % ID | % Coverage | BLAST Score | E-value |
| --- | --- | --- | --- | --- | --- | --- | --- |
| AAF08801.1 | YngI | BGC0001103 | Mycosubtilin\_biosynthetic\_gene\_cluster | 26 | 46.6010733453 | 155 | 4e-37 |
| AEW31026.1 | short-chain\_acyl-CoA\_synthetase | BGC0000407 | Plipastatin\_biosynthetic\_gene\_cluster | 26 | 46.6905187835 | 154 | 6.8e-37 |
| AAN32979.1 | BarE | BGC0000962 | Barbamide\_biosynthetic\_gene\_cluster | 25 | 44.9016100179 | 115 | 3.5e-25 |
| AHH25585.1 | AMP-dependent\_synthetase\_and\_ligase | BGC0000957 | Ansatrienin\_(mycotrienin)\_biosynthetic\_gene\_cluster | 25 | 44.8121645796 | 112 | 3e-24 |
| AEC14349.1 | nonribosomal\_peptide\_synthetase | BGC0000377 | Koranimine\_biosynthetic\_gene\_cluster | 24 | 56.7084078712 | 102 | 3.1e-21 |
| BAD55612.1 | non-ribosomal\_peptide\_synthetase | BGC0001027 | Nocobactin\_NA\_biosynthetic\_gene\_cluster | 22 | 54.114490161 | 74 | 1.2e-12 |
| AFR69331.1 | nonribosomal\_peptide\_synthetase\_SpiA | BGC0001045 | Spiruchostatin\_biosynthetic\_gene\_cluster | 22 | 34.9731663685 | 62 | 2.7e-09 |
| CCC21111.1 | putative\_peptide\_synthetase | BGC0000171 | 9-methylstreptimidone\_biosynthetic\_gene\_cluster | 24 | 38.9982110912 | 54 | 9.5e-07 |
